# Dr. William Henry Smith

**Published:** 1897-08

**Authors:** 


### Obituary.

### DR. WILLIAM HENRY SMITH.

Dr. William Henry Smith died at Newport, R. I., June 23, 1897, of senile asthenia and valvular insufficiency, in the eightieth year of his age.

Dr. Smith was born in Pawtuxet, R. I., December 1, 1817. He studied dentistry under a preceptor in Norwich, Conn., and began practice in Newport, R. I., nearly fifty years ago, continuing in the work there up to the time of his last sickness. He was one of the oldest practising dentists in the State, and his working-hours during his most active period were decidedly the larger part of the twenty-four, but the time was spent to the satisfaction of his patients. He displayed some mechanical ingenuity by inventions outside of his profession.

A married daughter, two grandchildren, and three great-grand-children survive him.

